# Decompression and Enucleation of a Mandibular Radicular Cyst, Followed by Bone Regeneration and Implant-Supported Dental Restoration

**DOI:** 10.1155/2019/9584235

**Published:** 2019-01-09

**Authors:** M. AboulHosn, Z. Noujeim, N. Nader, A. Berberi

**Affiliations:** ^1^Department of Oral & Maxillofacial Surgery, Faculty of Dental Medicine, Lebanese University, Beirut, Lebanon; ^2^Professor and Head Department of Oral & Maxillofacial Surgery, Faculty of Dental Medicine, Lebanese University, Beirut, Lebanon

## Abstract

Odontogenic cysts are usually treated by enucleation (cystectomy). Limited cysts (less than 5 cm) are usually managed by primary excision (total cystectomy), whereas larger ones (exceeding 5 cm) are often decompressed or marsupialized. Because it consists only of opening a much smaller surgical window, decompression is regarded as a more conservative method of treatment: this method associates the creation of an opening (window) into the cystic cavity with the suturing of a decompressing device (plastic tube or stent) at the periphery of the cyst. Apart from releasing intraluminal pressure in the pathological cavity, this procedure helps the lesion to progressively decrease in volume “with a gradual increase in bone apposition” and preserves pulp vitality and periodontal integrity of the adjacent teeth. We are reporting a case of a mandibular radicular cyst that was treated by decompression, followed by enucleation, bone reconstruction, and restoration with two osseointegrated dental implants. The cystic cavity progressively decreased in volume and increased in bone density.

## 1. Introduction

Marsupialization of odontogenic cystic lesions was described by Partsch in 1892; it is a technique where a large window is made in cystic wall and then sutured to the oral mucosa [1]. Decompression, proposed by Thoma [2], can be performed by using devices such as tube or stent [3], it's based on creation a window between the cyst and the oral cavity by fixing the device [4]. The decompression procedure may be easier to perform and more conservative than marsupialization [3, 4].

Bone defects can occur inevitably after mandibular cyst enucleation. A bone graft can be planned to promote bone regeneration of this bony defect [5, 6]. The remaining cystic cavity can be filled with different grafting materials to accelerate bone healing and anatomical regeneration and provide adequate support to the teeth and alveolar bone [2, 7, 8].

The aim of the present study is to present a clinical case report of a radicular cyst in a female patient that was treated by decompression, followed by cyst's enucleation, bone reconstruction of the cyst cavity, and dental implant placement and restoration.

## 2. Case Report

A 45-year-old female patient consulted our office with a chief complaint of left facial swelling and numbness of her chin, left lower lip, and a part of her left buccal mucosa and mandibular posterior vestibule. The patient had a history of endodontic treatment of her mandibular second left premolar (tooth 35) that was not accomplished by her restorative dentist; she also revealed that the swelling increased and reached her mid lower face, extending to the left anterior triangle of her neck, as well. She was treated by her restorative dentist with intramuscular injections of Rocephin® (ceftriaxone) during 4 weeks without remission.

Patient originally consulted for left facial swelling and paresthesia of left lower lip and chin. The left posterior mandibular vestibule, and particularly apical regions of 34 to 37, was spontaneously painful.

Intraoral examination displayed two swellings (in the left mandibular vestibule in relation to teeth 35 and 36) that were tender to palpation. On clinical examination, teeth 35 and 36 showed extreme mobility and were painful on horizontal and vertical percussion. Tooth 35 was open to access pulpal cavity, and thermal pulp test was negative on teeth 34 and 36 and positive on tooth 37.

**Figure 1:**
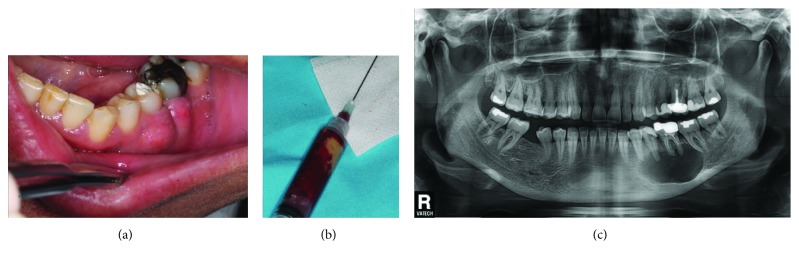
(a) Clinical aspect of left mandibular vestibule (premolar and molar regions). (b) Aspiration syringe yielding blood and pus from the radiolucent image. (c) OPG of the patient displaying the radiolucent image in left mandible (34 to 37).

An orthopantomogram (OPG) was taken in order to determine a possible bony pathology underneath the swollen left mandibular vestibule: OPG displayed a well-limited radiolucent image, extending from tooth 34 to tooth 37 and occupying most of the mandibular basal bone, in this sector. Periapical radiolucent lesion measured around 3-4 cm in length with around 2 cm width. It appeared unilocular, with well-defined, nonsclerotic borders, extending from mesial aspect of 34 to mesial aspect of mesial root of tooth 37, and almost reaching lower border of the mandible. Aspiration of the lesion was performed under local analgesia and it released pus and blood and a presumptive diagnosis of infected radicular cyst was made (Figures 1(a)–1(c)).

**Figure 2:**
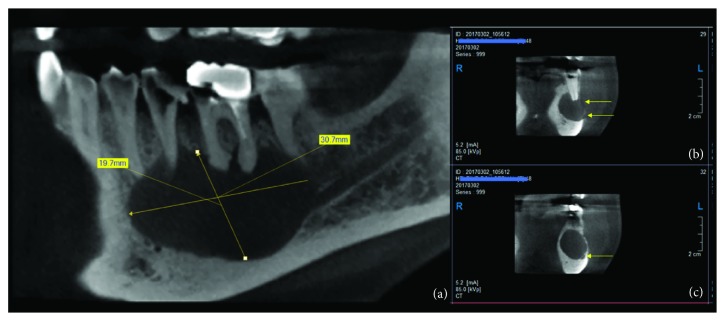
CBCT reconstruction of the left mandible. (a) The sagittal reconstruction with the approximative measurement of the bone destruction (30.7 mm × 19.7 mm). (b) Para-axial cut showing the discontinuity of the buccal cortical bone. (c) Para-axial cut showing the position of the mandibular canal.

Axial and coronal cuts of a cone beam computed tomography (CBCT) showed a radiolucency, ball shaped, measuring approximately 30.7 mm × 19.7 mm with discontinuity of the buccal wall, and the mandibular canal is repressed toward the basic bone of the mandible (Figures 2(a)–2(c)).

Treatment plan was decided and discussed with the patient, and the agreement was to undertake root canal treatment of tooth 34 and extract teeth 35 and 36 and to perform decompression of the radiolucent lesion through their alveolar cavities, after extractions of these teeth.

Block analgesia of the left inferior alveolar nerve was implemented with block 2% articaine with 1 : 100,000 adrenaline (3M ESPE, Seefeld, Germany); teeth 35 and 36 were extracted using elevators and forceps.

**Figure 3:**
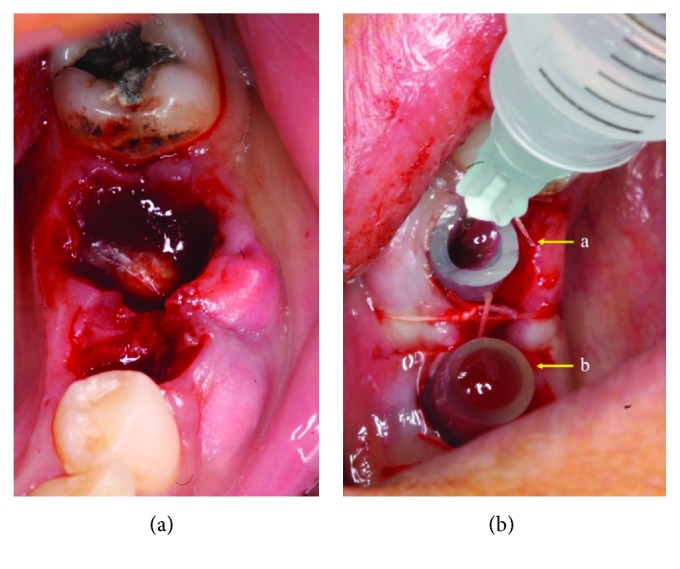
(a) Clinical aspect of alveolar cavities of 35 and 36, immediately after their extraction. (b) Plastic tubes secured in alveolar cavities of teeth 35 and 36 with sutures. A disposable syringe (with needle) injecting a normal saline solution (sodium chloride in water) in distal tube in order to regularly irrigate the lesions. After injection of antiseptic solution in distal tube (A), irrigating liquid is evacuated from mesial tube (B).

An incision through alveolar processes of 35 and 36 was made with no. 15 blade and the lesion's lining was fenestrated with a scalpel's blade: two transparent plastic tubes, sectioned from the tubing of a standard saline bag, were inserted in each alveolar cavity, and two interrupted sutures, from each side of the tubes, were placed to stabilize them (Figures 3(a) and 3(b)).

The patient was instructed to self-irrigate the lesion with a plastic disposable syringe with needle, with two antiseptic solutions, normal saline solution (0.9% sodium chloride in water) during the day (once every 3 hours and after each meal) and 0.12% chlorhexidine gluconate in the morning and in the evening before bedtime.

**Figure 4:**
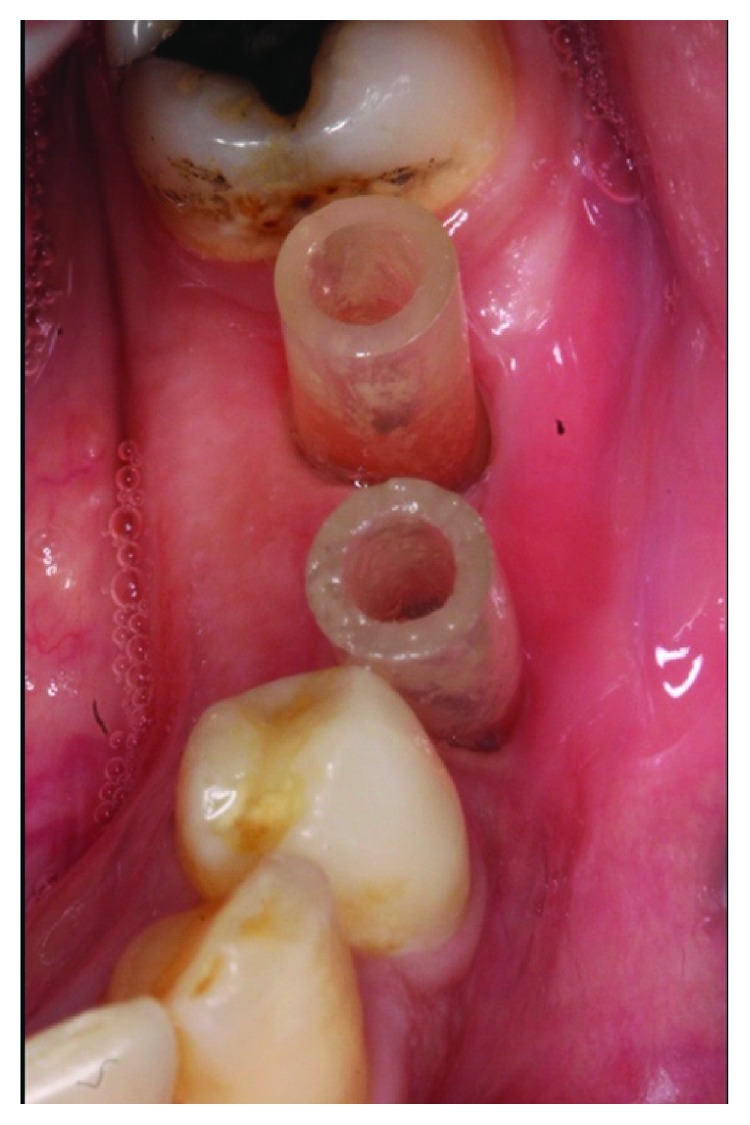
Gingival healing around decompressing devices (tubes), 3 weeks after their placement and suturing.

The patient was recalled every week for irrigation of the lesion and check-up. What is special in this irrigation modality is that the antiseptic solution (saline and/or chlorhexidine gluconate) was injected through one tube and drained from the other one, instantaneously. At biweekly intervals, the length of the tube was monitored and cut in order to keep it far from occlusion (Figure 4).

**Figure 5:**
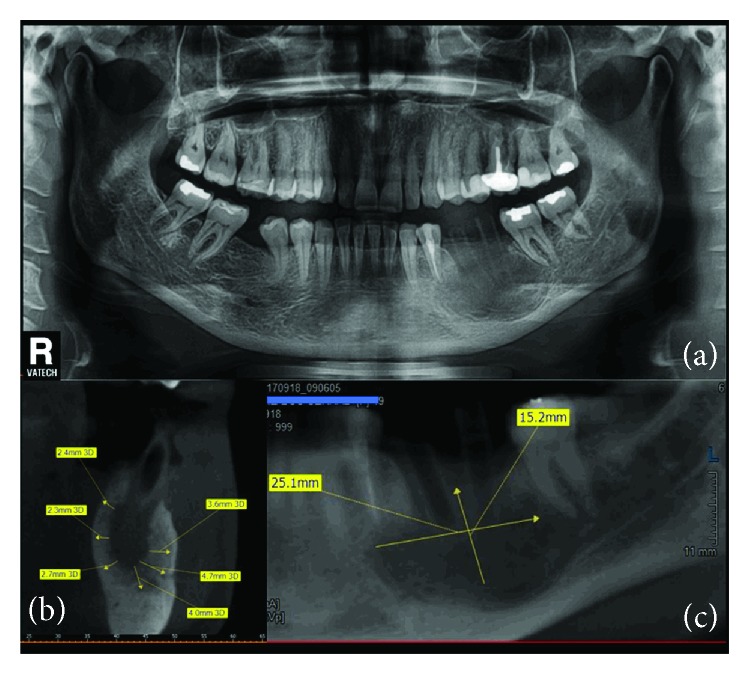
(a) Panoramic radiograph 7 months after decompression, showing a reduction of the volume of the lesion. (b) CBCT of the same time period time, the para-axial cut shows a bone healing toward the center of the lesions with approximately ±4 mm buccally and ±2.4 mm lingually. (c) Sagittal reconstruction of the CBCT shows a volume reduction of the lesion approximately.

The lesion's enucleation was planned to be implemented 6 months after decompression that would reduce its size. Indeed, 6 months after the first lesion's irrigation, a panoramic radiograph was taken in order to evaluate the bone apposition: bone formation took place within the lesion as observed on CBCT performed after 7 months, with an almost complete formation of buccal cortical and bone apposition buccally (±4 mm) and lingually (±2.4 mm) as shown on the para-axial cut and lesion volume reduction as shown on the sagittal reconstruction (±25.1 mm × 15.2 mm), all along the lesion (Figures 5(a)–5(c)).

At first week of the 7th postoperative month, the decompression tubes were removed. With a blade #15, a crestal incision followed by 2 relaxing incisions was performed before raising a full-thickness flap. After separating the lesion's membrane from the flap, enucleation was accomplished with a Lucas curette and the entire pathological specimen was collected and immersed in a fixative solution (20% formol) for histopathological examination.

**Figure 6:**
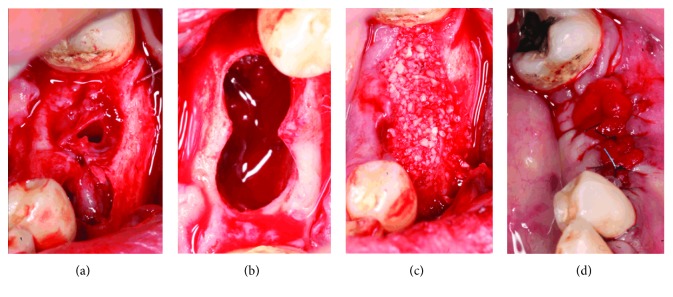
(a) Six months after the beginning of decompression, a mucoperiosteal flap was performed and decompression tubes removed, leading to the roof of the radiolucent lesion. (b) Upper aspect of the bony pathological cavity, immediately after the lesion's enucleation. Notice that the bone was formed all around external surfaces of both decompression devices (tubes). (c) The pathological cavity filled with Puros® cortico-cancellous particulate allograft, a mixture of 70% cortical and 30% cancellous bone particulate. (d) Puros® biomaterial covered with collagen cones (CollaPlug®) and mucosal wound sutured with 5/0 nylon sutures.

During operation, it was noticed that the buccal bone wall took the same shape of drainage tubes after reformation. After the lesion's enucleation, the bony cavity was filled with Puros® cortico-cancellous particulate allograft (Zimmer Biomet Dental, Carlsbad, California, USA) due to its osteoconductive properties. The flap was relocated with 5/0 nylon interrupted sutures, and tube opening locations were covered with collagen (CollaTape®) over bone graft (Figures 6(a)–6(d)).

**Figure 7:**
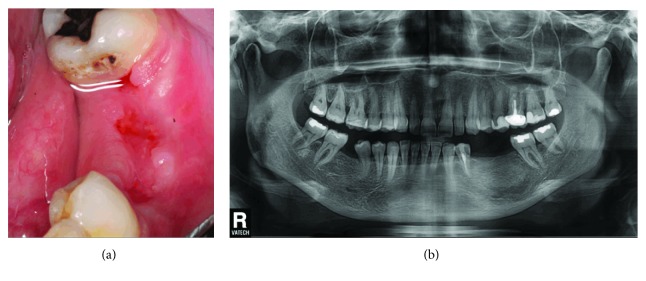
(a) Clinical mucosal healing after one week. (b) Radiological aspect of the graft and surrounding bone.

The patient consulted us again one week after the cyst enucleation with less than 20% of left lower lip/chin numbness remaining, and two weeks later, she almost recovered her normal sensation, with a mild left lower lip “heaviness” remaining (Figures 7(a) and 7(b)).

**Figure 8:**
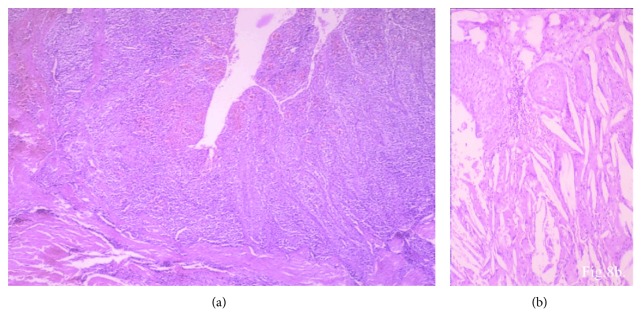
Histopathology of the cyst. (a) Stratified squamous nonkeratinized epithelium with rete ridges and inflamed connective tissue wall (H&E × 20). (b) Cystic lumen is filled with liquid containing cholesterol crystals (H&E × 40). Mucoperiosteal flap showing the buccolingual thickness of mandibular ridge before implant placement.

Pathological report concluded that the lesion was a radicular cyst (Figure 8).

**Figure 9:**
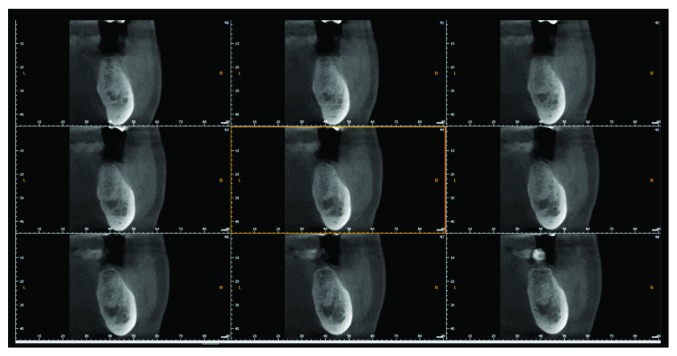
Para-axial cuts of a control CBCT before implant placement showing a complete bone regeneration of the cyst area.

**Figure 10:**
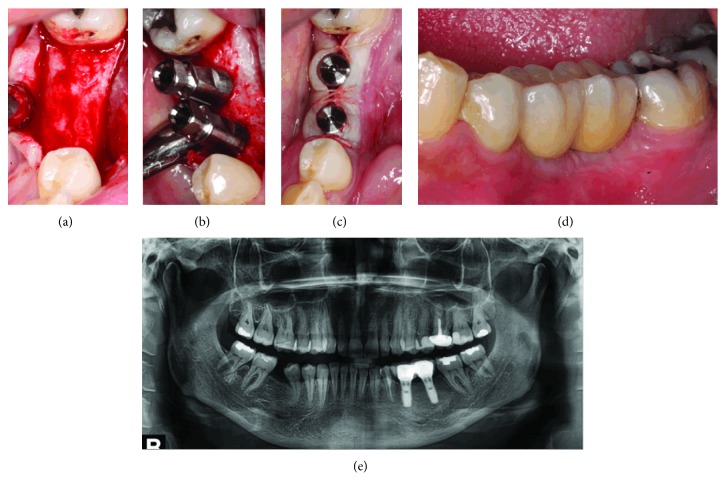
(a) Clinical view of the bone healing before implant placement. (b) Two Swiss Plus Zimmer® implants placed in postalveolar locations of teeth 35 and 36. (c) Clinical aspects of cover screws and sutures, immediately after implants placement. (d) Clinical aspect of restored implants (35 and 36). (e) Panoramic radiograph showing the final situation of the bone and the restored implants.

Six months following bone grafting, CBCT shows a complete bone regeneration of the whole area (Figure 9) and two SwissPlus Zimmer® implants were placed in locations of teeth 35 and 36 and were restored with cemented prostheses 3 months after their surgical placement (Figures 10(a)–10(e)).

## 3. Discussion

Radicular cyst is the most frequent odontogenic cyst observed in tooth-bearing areas: it is usually associated with carious, nonvital, discolored, or fractured tooth. Dental caries are known to provocate dental pulp inflammation that leads, ultimately, to pulp necrosis; infection will then spread to periradicular space, causing periodontitis that usually precedes either periradicular acute abscess, chronic granuloma, or radicular cyst. This cyst usually follows persistent chronic infection, and it is believed to form by proliferation of the epithelial cell rests of Malassez in inflamed periradicular tissues [9].

Several treatment options are nowadays available for a radicular cyst such as nonsurgical root canal therapy (endodontic treatment), extraction of the offending tooth if unrestorable, decompression, marsupialization, and enucleation with primary closure, when the lesion is large [6]. The treatment of choice depends on the size and localization of the lesion, integrity of the cystic epithelial lining, proximity of the cyst to adjacent vital teeth and anatomical structures (such as inferior alveolar canal, mental foramen, infraorbital foramen, maxillary sinus, nasal cavity, and infratemporal space), and behavior of the cyst (clinical aggressively and radiological invasiveness) [10].

Decompression procedure is “an alternative plan of treatment and a more conservative approach; this procedure allows for continuous drainage, which removes those conditions that favor cyst expansion” [8]. In the present case report, an aspiration of a serosanguinous purulent fluid confirmed the provisional, presumptive diagnosis of a “cystic” lesion (presumptive radicular cyst), knowing that cystic content may be straw colored, cloudy, or serosanguinous. Failure to withdraw any kind of intracystic fluid or repeated aspirations of blood warrant a reevaluation of the provisional diagnosis and absence of any aspirate liquid may suggest a solid cellular lesion of neoplastic origin or a fibrous one [11].

Decompression devices (tubes) aim to maintain drainage of jaw pathological lesions and facilitate their repeated irrigations [4]: in our case, fabrication of these tubes consisted of a section of the tubing from a standard saline solution bag. We decided to use this kind of tubing because it is not collapsible, readily available, and sufficient in diameter to prevent clogging; they can be adjusted and fashioned for patient comfort as well and their insertion and removal are easy and appropriate.

Decompression tubes were immersed in 0.12% chlorhexidine gluconate for 15 minutes and then sterilized before placement in the pathological cavity. They were inserted using a slight rotary motion and gently pushed downwards into the depth of the lesion until resistance was met at the lesion's lower level. Initial drainage occurred immediately after their insertion.

It is widely known that decompression and marsupialization are conservative treatment modalities that involve an opening to reduce intracystic pressure and induce bony formation [2, 3, 12]: both procedures provide good surgical access to lesions with low postoperative morbidity and low incidence of peroperative complications [13, 14], help in maintaining pulp vitality of intracystic teeth, shrink the lesions' volume, and reduce their recurrence rates [15, 16].

In our case, decompression led to reduce the volume of the cyst from 30.7 mm × 19.7 mm to 25.1 mm × 15.2 mm approximately. The CBCT shows a complete bone formation of the cortical buccal plate and bone apposition from the buccal bone and lingual bone in a central direction measured as ±4 mm and ±2.4 respectively.

The effects of decompression based on age are controversial: it was reported that younger patients had higher reduction rates [12], but it was also known that decompression and incidence of recurrence are not correlated with age [17].

Another advantage of these two methods, particularly decompression, is the positive effect they have on the histological nature of the cyst's epithelial lining. In a study on 14 decompressed odontogenic keratocysts (OKCs) with 65% of mean shrinkage of the radiolucency, August and coworkers reported a dedifferentiation and loss of cytokeratin-10 production in 64% of decompression and irrigation patients after a 9-month average treatment time: changes in cystic epithelium appeared to be a gradual process and their results suggested that a treatment time of at least 9 months may be required to induce epithelial dedifferentiation [18].

Schlieve and coworkers [19] studied the possible histological diagnostic changes after decompression of odontogenic cysts and cyst-like lesions: the purpose of their study was to report the histopathological findings after postdecompression definitive treatment, thereby answering the clinical question “does decompression change the histologic diagnosis?” Twenty-five cysts and cyst-like lesions in 25 patients were decompressed before being enucleated and curetted. Postdecompression histological examination at the time of definitive surgical treatment was found consistent with the preoperative biopsy diagnosis in 91% of keratocystic odontogenic tumors, 67% of glandular odontogenic cysts, 75% of dentigerous cysts, and 100% of cystic ameloblastomas. Authors concluded that all decompressed lesions should be definitely treated based on the initial diagnosis, with all patients placed on an appropriate follow-up protocol dependent on the original diagnosis [19]. However, other authors found no statistical evidence that decompression influences expression of proliferation markers in the lining, indicating that the potential for recurrence may not be restricted to cellular level [20, 21].

The most important requisites for bone deposition in a bone cavity includes the presence of blood clot, a source of osteoblasts, a contact with living tissue, and prevention of soft tissue invasion [22]. There have been controversies regarding bony regeneration in cystic cavities with or without bone grafts [23]. Some authors reported treating cyst cavities without using any graft materials and found new bone apposition after a period of one year [24, 25]. Others have reported using resorbable and nonresorbable grafts for bone defects secondary to the removal of cysts with good outcomes [26–28].

In our case, after the cyst's enucleation, we grafted the cavity with allograft, an osteoconductive material [29], knowing that osteoconductive materials are thought to stabilize blood clot and advance bone regeneration providing a scaffold, thus enhancing the migration of osteoprogenitor cells [8]. Without filling the cavity, we believe that there would have been tissue collapse in the cavity. The bone regeneration was observed after 6 months and allowed to place dental implants with a good primary stability.

## 4. Conclusion

Radicular cyst is commonly found in the jaws. This case report illustrates the successful management of a mandibular radicular cyst by decompression, enucleation, grafting, and site rehabilitation.
